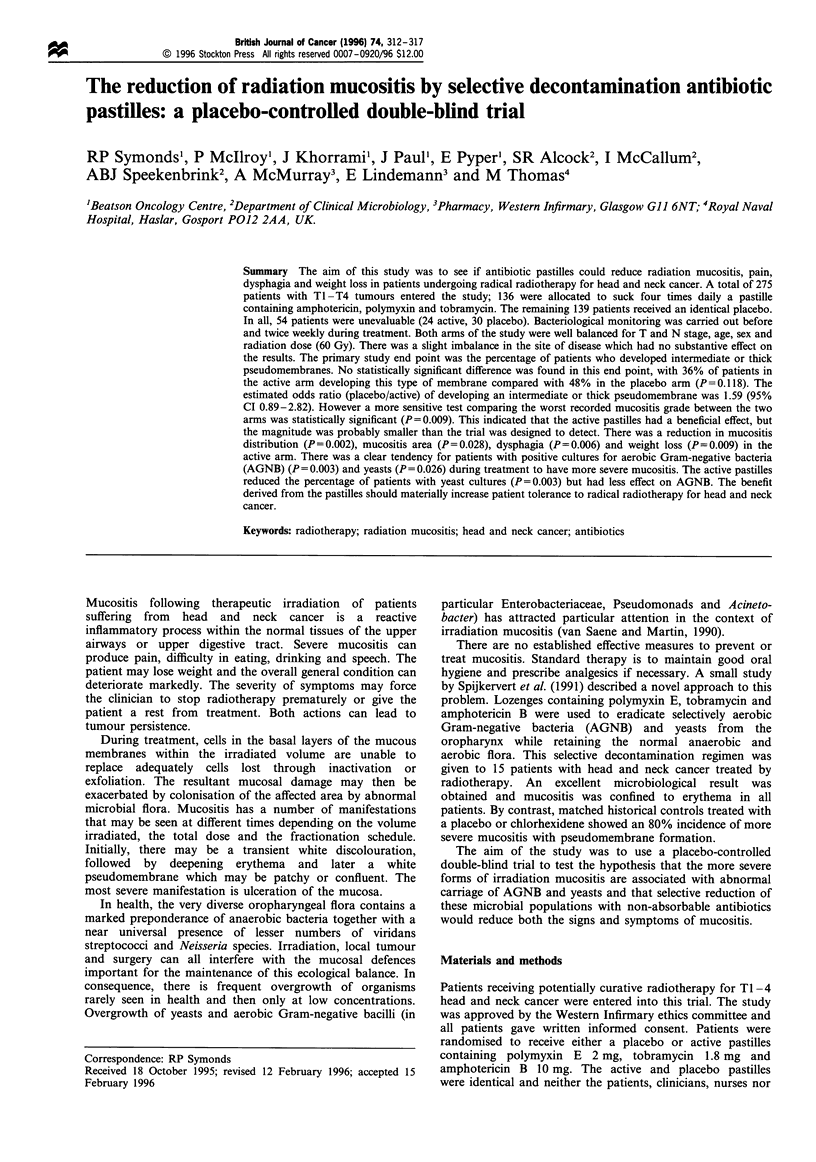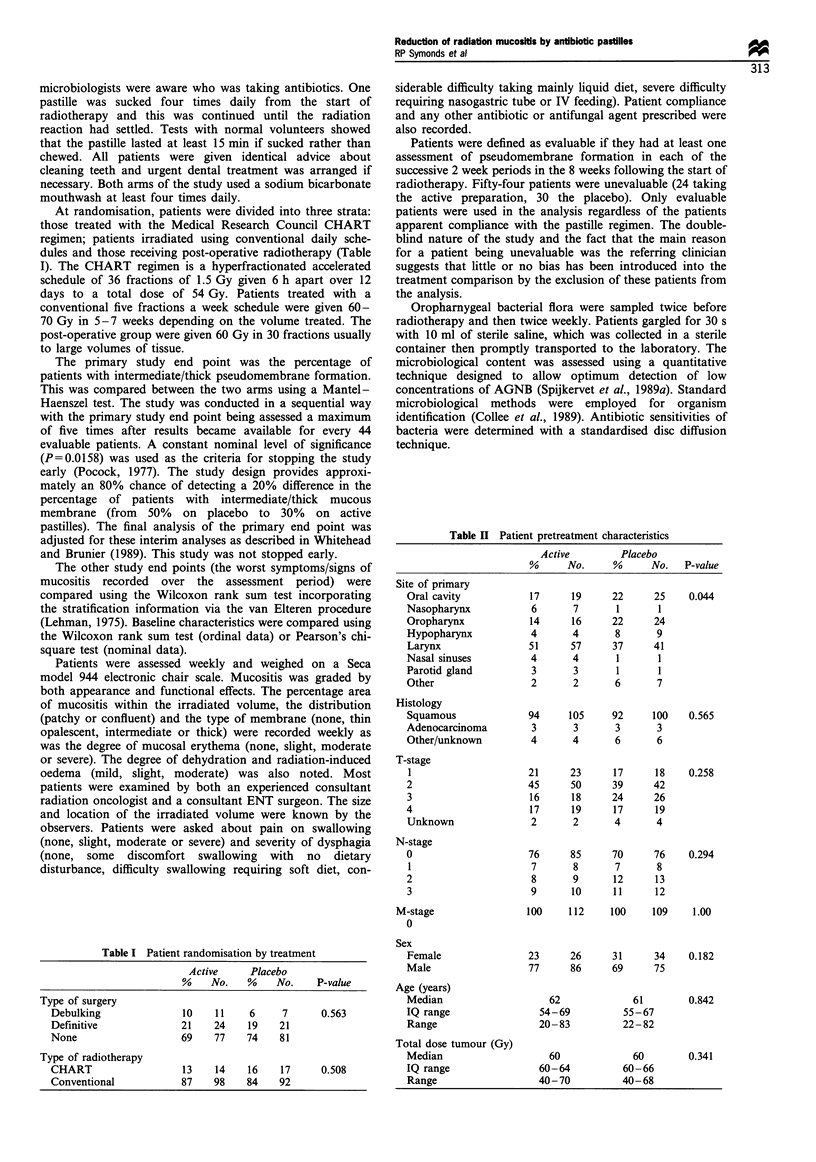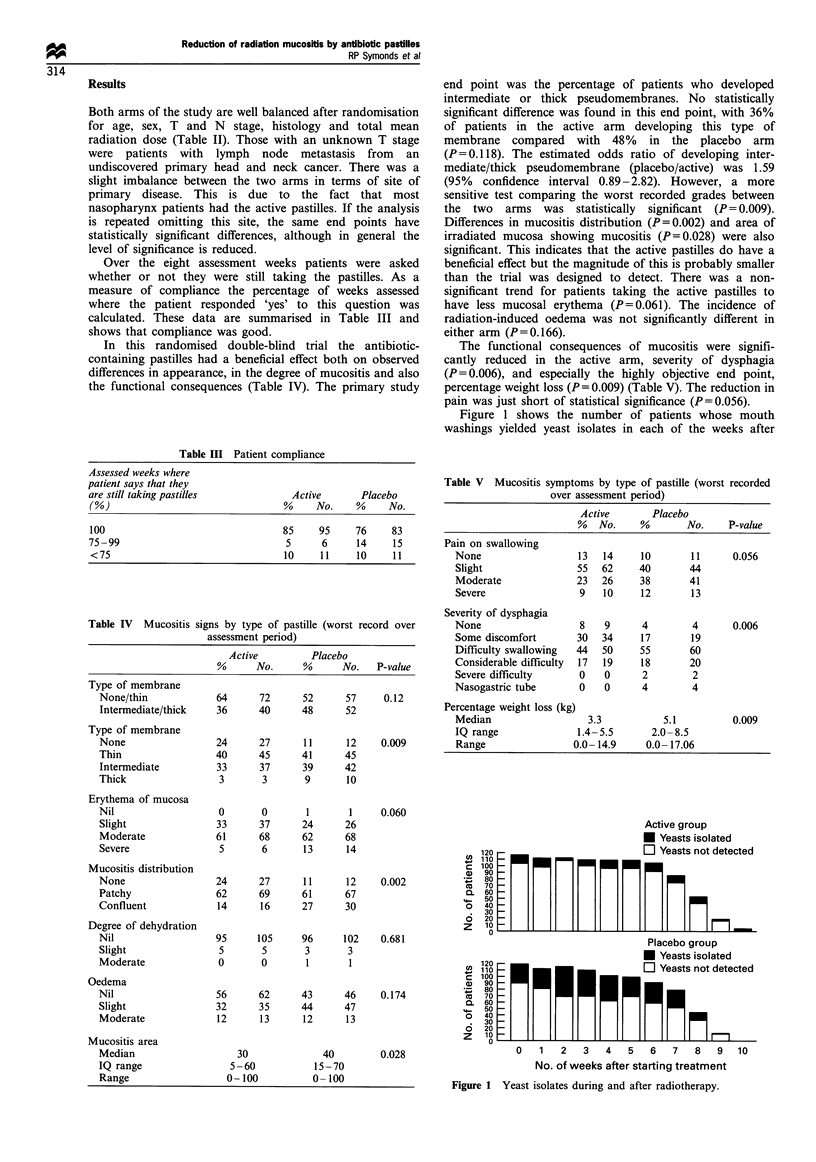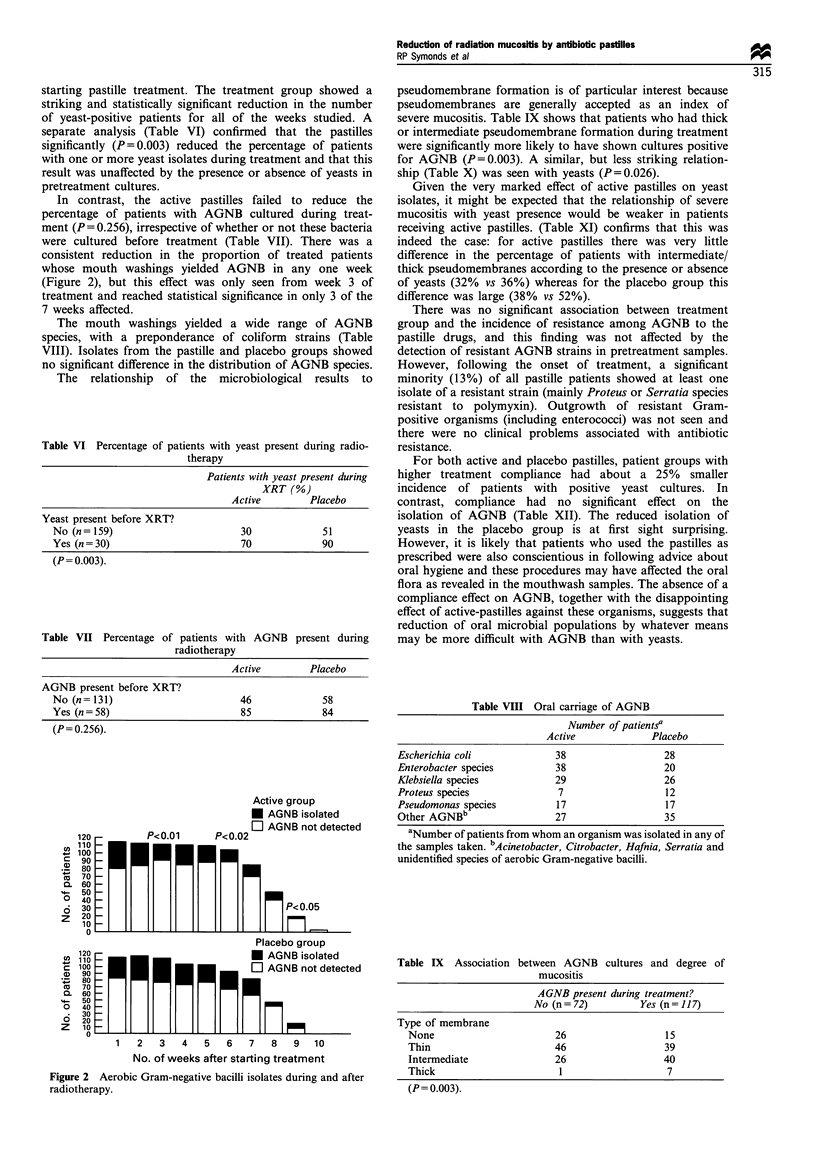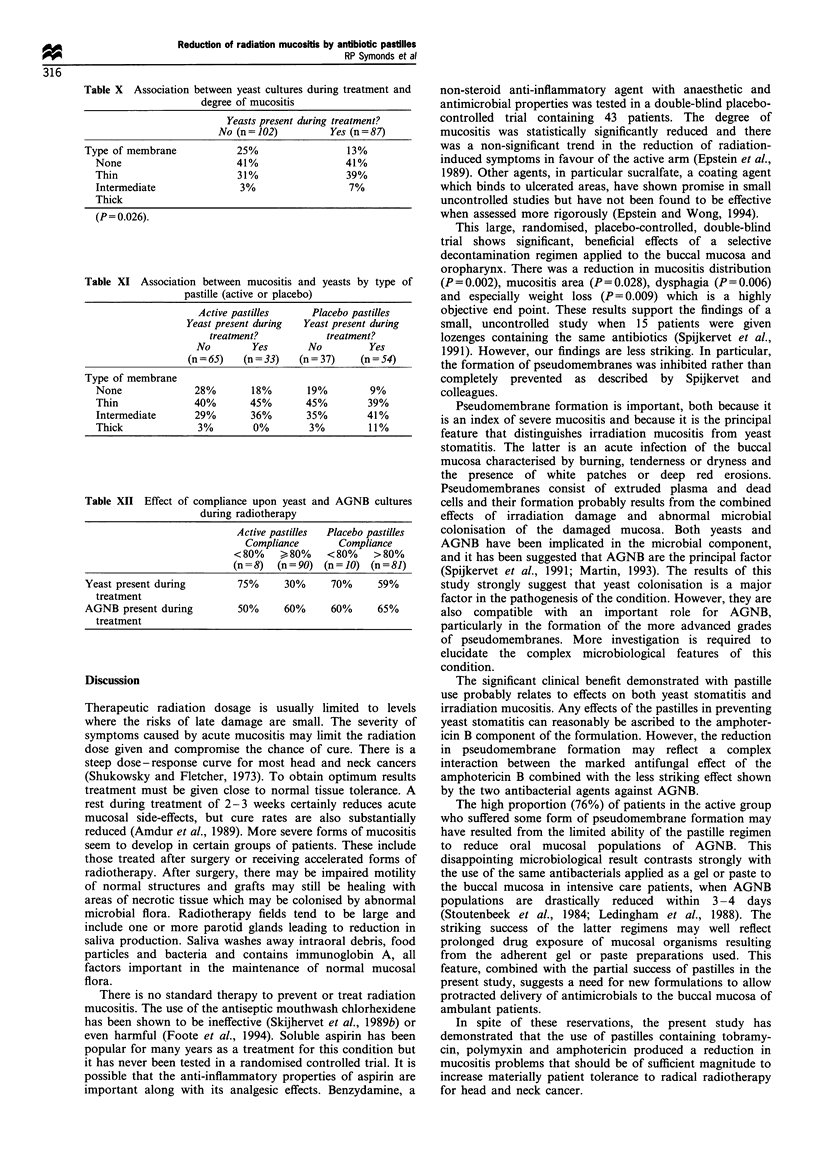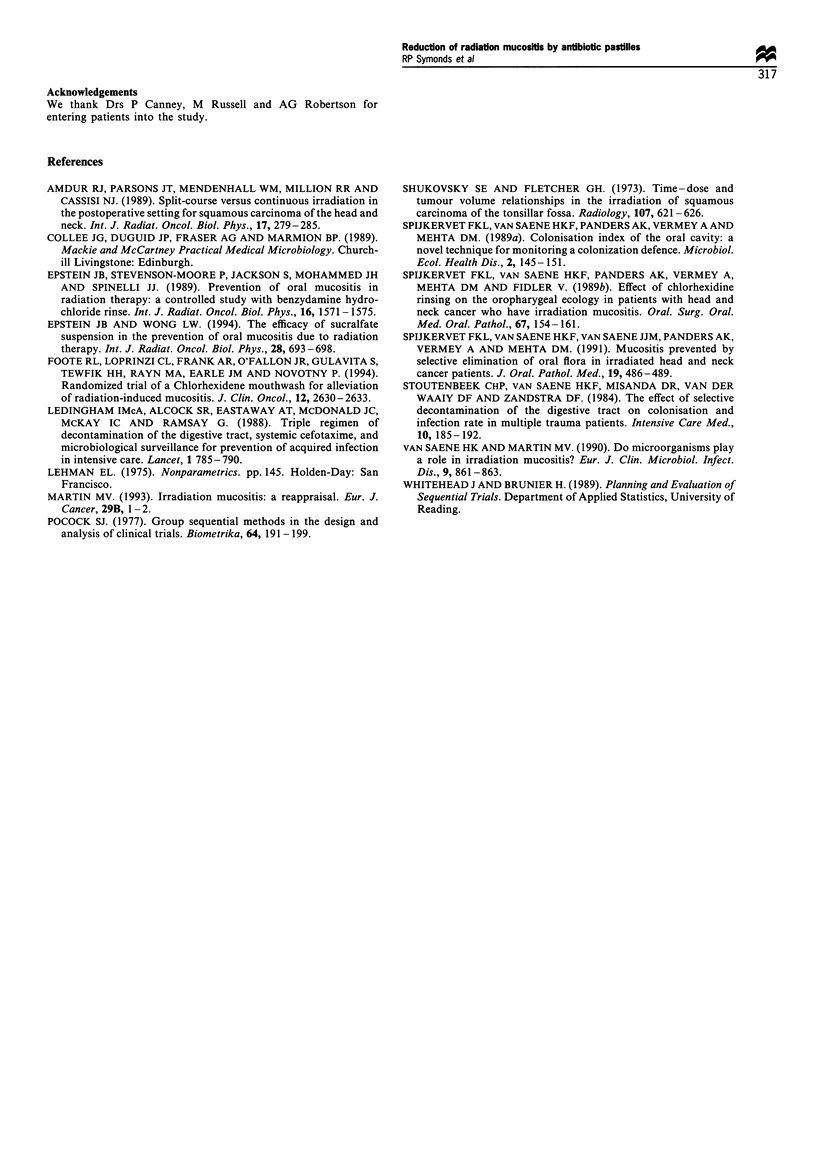# The reduction of radiation mucositis by selective decontamination antibiotic pastilles: a placebo-controlled double-blind trial.

**DOI:** 10.1038/bjc.1996.359

**Published:** 1996-07

**Authors:** R. P. Symonds, P. McIlroy, J. Khorrami, J. Paul, E. Pyper, S. R. Alcock, I. McCallum, A. B. Speekenbrink, A. McMurray, E. Lindemann, M. Thomas

**Affiliations:** Beatson Oncology Centre, Western Infirmary, Glasgow, UK.

## Abstract

The aim of this study was to see if antibiotic pastilles could reduce radiation mucositis, pain, dysphagia and weight loss in patients undergoing radical radiotherapy for head and neck cancer. A total of 275 patients with T1-T4 tumours entered the study; 136 were allocated to suck four times daily a pastille containing amphotericin, polymyxin and tobramycin. The remaining 139 patients received an identical placebo. In all, 54 patients were unevaluable (24 active, 30 placebo). Bacteriological monitoring was carried out before and twice weekly during treatment. Both arms of the study were well balanced for T and N stage, age, sex and radiation dose (60 Gy). There was a slight imbalance in the site of disease which had no substantive effect on the results. The primary study end point was the percentage of patients who developed intermediate or thick pseudomembranes. No statistically significant difference was found in this end point, with 36% of patients in the active arm developing this type of membrane compared with 48% in the placebo arm (P = 0.118). The estimated odds ratio (placebo/active) of developing an intermediate or thick pseudomembrane was 1.59 (95% CI 0.89-2.82). However a more sensitive test comparing the worst recorded mucositis grade between the two arms was statistically significant (P = 0.009). This indicated that the active pastilles had a beneficial effect, but the magnitude was probably smaller than the trial was designed to detect. There was a reduction in mucositis distribution (P = 0.002), mucositis area (P = 0.028), dysphagia (P = 0.006) and weight loss (P = 0.009) in the active arm. There was a clear tendency for patients with positive cultures for aerobic Gram-negative bacteria (AGNB) (P = 0.003) and yeasts (P = 0.026) during treatment to have more severe mucositis. The active pastilles reduced the percentage of patients with yeast cultures (P = 0.003) but had less effect on AGNB. The benefit derived from the pastilles should materially increase patient tolerance to radical radiotherapy for head and neck cancer.


British Journal of Cancer (1996) 74, 312-317
© 1996 Stockton Press All rights reserved 0007-0920/96 $12.00

### The reduction of radiation mucositis by selective decontamination antibiotic pastilles: a placebo-controlled double-blind trial

RP Symonds[1], P McIlroy[1], J Khorrami[1], J Paul[1], E Pyper[1], SR Alcock[2], I McCallum[2], ABJ Speekenbrink[2], A McMurray[3], E Lindemann[3] and M Thomas[4]

*[1]Beatson Oncology Centre, [2]Department of Clinical Microbiology, [3]Pharmacy, Western Infirmary, Glasgow G11 6NT; [4]Royal Naval Hospital, Haslar, Gosport PO12 2AA, UK.*

**Summary** The aim of this study was to see if antibiotic pastilles could reduce radiation mucositis, pain, dysphagia and weight loss in patients undergoing radical radiotherapy for head and neck cancer. A total of 275 patients with T1-T4 tumours entered the study; 136 were allocated to suck four times daily a pastille containing amphotericin, polymyxin and tobramycin. The remaining 139 patients received an identical placebo. In all, 54 patients were unevaluable (24 active, 30 placebo). Bacteriological monitoring was carried out before and twice weekly during treatment. Both arms of the study were well balanced for T and N stage, age, sex and radiation dose (60 Gy). There was a slight imbalance in the site of disease which had no substantive effect on the results. The primary study end point was the percentage of patients who developed intermediate or thick pseudomembranes. No statistically significant difference was found in this end point, with 36% of patients in the active arm developing this type of membrane compared with 48% in the placebo arm ($P=0.118$). The estimated odds ratio (placebo/active) of developing an intermediate or thick pseudomembrane was 1.59 (95% CI 0.89-2.82). However a more sensitive test comparing the worst recorded mucositis grade between the two arms was statistically significant ($P=0.009$). This indicated that the active pastilles had a beneficial effect, but the magnitude was probably smaller than the trial was designed to detect. There was a reduction in mucositis distribution ($P=0.002$), mucositis area ($P=0.028$), dysphagia ($P=0.006$) and weight loss ($P=0.009$) in the active arm. There was a clear tendency for patients with positive cultures for aerobic Gram-negative bacteria (AGNB) ($P=0.003$) and yeasts ($P=0.026$) during treatment to have more severe mucositis. The active pastilles reduced the percentage of patients with yeast cultures ($P=0.003$) but had less effect on AGNB. The benefit derived from the pastilles should materially increase patient tolerance to radical radiotherapy for head and neck cancer.

**Keywords:** radiotherapy; radiation mucositis; head and neck cancer; antibiotics

Mucositis following therapeutic irradiation of patients suffering from head and neck cancer is a reactive inflammatory process within the normal tissues of the upper airways or upper digestive tract. Severe mucositis can produce pain, difficulty in eating, drinking and speech. The patient may lose weight and the overall general condition can deteriorate markedly. The severity of symptoms may force the clinician to stop radiotherapy prematurely or give the patient a rest from treatment. Both actions can lead to tumour persistence.

During treatment, cells in the basal layers of the mucous membranes within the irradiated volume are unable to replace adequately cells lost through inactivation or exfoliation. The resultant mucosal damage may then be exacerbated by colonisation of the affected area by abnormal microbial flora. Mucositis has a number of manifestations that may be seen at different times depending on the volume irradiated, the total dose and the fractionation schedule. Initially, there may be a transient white discolouration, followed by deepening erythema and later a white pseudomembrane which may be patchy or confluent. The most severe manifestation is ulceration of the mucosa.

In health, the very diverse oropharyngeal flora contains a marked preponderance of anaerobic bacteria together with a near universal presence of lesser numbers of viridans streptococci and *Neisseria* species. Irradiation, local tumour and surgery can all interfere with the mucosal defences important for the maintenance of this ecological balance. In consequence, there is frequent overgrowth of organisms rarely seen in health and then only at low concentrations. Overgrowth of yeasts and aerobic Gram-negative bacilli (in particular Enterobacteriaceae, Pseudomonads and *Acinetobacter*) has attracted particular attention in the context of irradiation mucositis (van Saene and Martin, 1990).

There are no established effective measures to prevent or treat mucositis. Standard therapy is to maintain good oral hygiene and prescribe analgesics if necessary. A small study by Spijkervert *et al.* (1991) described a novel approach to this problem. Lozenges containing polymyxin E, tobramycin and amphotericin B were used to eradicate selectively aerobic Gram-negative bacteria (AGNB) and yeasts from the oropharynx while retaining the normal anaerobic and aerobic flora. This selective decontamination regimen was given to 15 patients with head and neck cancer treated by radiotherapy. An excellent microbiological result was obtained and mucositis was confined to erythema in all patients. By contrast, matched historical controls treated with a placebo or chlorhexidene showed an 80% incidence of more severe mucositis with pseudomembrane formation.

The aim of the study was to use a placebo-controlled double-blind trial to test the hypothesis that the more severe forms of irradiation mucositis are associated with abnormal carriage of AGNB and yeasts and that selective reduction of these microbial populations with non-absorbable antibiotics would reduce both the signs and symptoms of mucositis.

### Materials and methods

Patients receiving potentially curative radiotherapy for T1-4 head and neck cancer were entered into this trial. The study was approved by the Western Infirmary ethics committee and all patients gave written informed consent. Patients were randomised to receive either a placebo or active pastilles containing polymyxin E 2 mg, tobramycin 1.8 mg and amphotericin B 10 mg. The active and placebo pastilles were identical and neither the patients, clinicians, nurses nor

Correspondence: RP Symonds
Received 18 October 1995; revised 12 February 1996; accepted 15 February 1996

microbiologists were aware who was taking antibiotics. One pastille was sucked four times daily from the start of radiotherapy and this was continued until the radiation reaction had settled. Tests with normal volunteers showed that the pastille lasted at least 15 min if sucked rather than chewed. All patients were given identical advice about cleaning teeth and urgent dental treatment was arranged if necessary. Both arms of the study used a sodium bicarbonate mouthwash at least four times daily.

At randomisation, patients were divided into three strata: those treated with the Medical Research Council CHART regimen; patients irradiated using conventional daily schedules and those receiving post-operative radiotherapy (Table I). The CHART regimen is a hyperfractionated accelerated schedule of 36 fractions of 1.5 Gy given 6 h apart over 12 days to a total dose of 54 Gy. Patients treated with a conventional five fractions a week schedule were given 60–70 Gy in 5–7 weeks depending on the volume treated. The post-operative group were given 60 Gy in 30 fractions usually to large volumes of tissue.

The primary study end point was the percentage of patients with intermediate/thick pseudomembrane formation. This was compared between the two arms using a Mantel–Haenszel test. The study was conducted in a sequential way with the primary study end point being assessed a maximum of five times after results became available for every 44 evaluable patients. A constant nominal level of significance ($P = 0.0158$) was used as the criteria for stopping the study early (Pocock, 1977). The study design provides approximately an 80% chance of detecting a 20% difference in the percentage of patients with intermediate/thick mucous membrane (from 50% on placebo to 30% on active pastilles). The final analysis of the primary end point was adjusted for these interim analyses as described in Whitehead and Brunier (1989). This study was not stopped early.

The other study end points (the worst symptoms/signs of mucositis recorded over the assessment period) were compared using the Wilcoxon rank sum test incorporating the stratification information via the van Elteren procedure (Lehman, 1975). Baseline characteristics were compared using the Wilcoxon rank sum test (ordinal data) or Pearson's chi-square test (nominal data).

Patients were assessed weekly and weighed on a Seca model 944 electronic chair scale. Mucositis was graded by both appearance and functional effects. The percentage area of mucositis within the irradiated volume, the distribution (patchy or confluent) and the type of membrane (none, thin opalescent, intermediate or thick) were recorded weekly as was the degree of mucosal erythema (none, slight, moderate or severe). The degree of dehydration and radiation-induced oedema (mild, slight, moderate) was also noted. Most patients were examined by both an experienced consultant radiation oncologist and a consultant ENT surgeon. The size and location of the irradiated volume were known by the observers. Patients were asked about pain on swallowing (none, slight, moderate or severe) and severity of dysphagia (none, some discomfort swallowing with no dietary disturbance, difficulty swallowing requiring soft diet, considerable difficulty taking mainly liquid diet, severe difficulty requiring nasogastric tube or IV feeding). Patient compliance and any other antibiotic or antifungal agent prescribed were also recorded.

Patients were defined as evaluable if they had at least one assessment of pseudomembrane formation in each of the successive 2 week periods in the 8 weeks following the start of radiotherapy. Fifty-four patients were unevaluable (24 taking the active preparation, 30 the placebo). Only evaluable patients were used in the analysis regardless of the patients apparent compliance with the pastille regimen. The double-blind nature of the study and the fact that the main reason for a patient being unevaluable was the referring clinician suggests that little or no bias has been introduced into the treatment comparison by the exclusion of these patients from the analysis.

Oropharnygeal bacterial flora were sampled twice before radiotherapy and then twice weekly. Patients gargled for 30 s with 10 ml of sterile saline, which was collected in a sterile container then promptly transported to the laboratory. The microbiological content was assessed using a quantitative technique designed to allow optimum detection of low concentrations of AGNB (Spijkervet *et al.*, 1989*a*). Standard microbiological methods were employed for organism identification (Collee *et al.*, 1989). Antibiotic sensitivities of bacteria were determined with a standardised disc diffusion technique.

**Table I** Patient randomisation by treatment

| | *Active* % | *Active* No. | *Placebo* % | *Placebo* No. | P-*value* |
|---|---|---|---|---|---|
| Type of surgery | | | | | |
| Debulking | 10 | 11 | 6 | 7 | 0.563 |
| Definitive | 21 | 24 | 19 | 21 | |
| None | 69 | 77 | 74 | 81 | |
| Type of radiotherapy | | | | | |
| CHART | 13 | 14 | 16 | 17 | 0.508 |
| Conventional | 87 | 98 | 84 | 92 | |

**Table II** Patient pretreatment characteristics

| | *Active* % | *Active* No. | *Placebo* % | *Placebo* No. | P-*value* |
|---|---|---|---|---|---|
| Site of primary | | | | | |
| Oral cavity | 17 | 19 | 22 | 25 | 0.044 |
| Nasopharynx | 6 | 7 | 1 | 1 | |
| Oropharynx | 14 | 16 | 22 | 24 | |
| Hypopharynx | 4 | 4 | 8 | 9 | |
| Larynx | 51 | 57 | 37 | 41 | |
| Nasal sinuses | 4 | 4 | 1 | 1 | |
| Parotid gland | 3 | 3 | 1 | 1 | |
| Other | 2 | 2 | 6 | 7 | |
| Histology | | | | | |
| Squamous | 94 | 105 | 92 | 100 | 0.565 |
| Adenocarcinoma | 3 | 3 | 3 | 3 | |
| Other/unknown | 4 | 4 | 6 | 6 | |
| T-stage | | | | | |
| 1 | 21 | 23 | 17 | 18 | 0.258 |
| 2 | 45 | 50 | 39 | 42 | |
| 3 | 16 | 18 | 24 | 26 | |
| 4 | 17 | 19 | 17 | 19 | |
| Unknown | 2 | 2 | 4 | 4 | |
| N-stage | | | | | |
| 0 | 76 | 85 | 70 | 76 | 0.294 |
| 1 | 7 | 8 | 7 | 8 | |
| 2 | 8 | 9 | 12 | 13 | |
| 3 | 9 | 10 | 11 | 12 | |
| M-stage 0 | 100 | 112 | 100 | 109 | 1.00 |
| Sex | | | | | |
| Female | 23 | 26 | 31 | 34 | 0.182 |
| Male | 77 | 86 | 69 | 75 | |
| Age (years) | | | | | |
| Median | 62 | | 61 | | 0.842 |
| IQ range | 54–69 | | 55–67 | | |
| Range | 20–83 | | 22–82 | | |
| Total dose tumour (Gy) | | | | | |
| Median | 60 | | 60 | | 0.341 |
| IQ range | 60–64 | | 60–66 | | |
| Range | 40–70 | | 40–68 | | |

### Results

Both arms of the study are well balanced after randomisation for age, sex, T and N stage, histology and total mean radiation dose (Table II). Those with an unknown T stage were patients with lymph node metastasis from an undiscovered primary head and neck cancer. There was a slight imbalance between the two arms in terms of site of primary disease. This is due to the fact that most nasopharynx patients had the active pastilles. If the analysis is repeated omitting this site, the same end points have statistically significant differences, although in general the level of significance is reduced.

Over the eight assessment weeks patients were asked whether or not they were still taking the pastilles. As a measure of compliance the percentage of weeks assessed where the patient responded 'yes' to this question was calculated. These data are summarised in Table III and shows that compliance was good.

In this randomised double-blind trial the antibiotic-containing pastilles had a beneficial effect both on observed differences in appearance, in the degree of mucositis and also the functional consequences (Table IV). The primary study end point was the percentage of patients who developed intermediate or thick pseudomembranes. No statistically significant difference was found in this end point, with 36% of patients in the active arm developing this type of membrane compared with 48% in the placebo arm ($P=0.118$). The estimated odds ratio of developing intermediate/thick pseudomembrane (placebo/active) was 1.59 (95% confidence interval 0.89–2.82). However, a more sensitive test comparing the worst recorded grades between the two arms was statistically significant ($P=0.009$). Differences in mucositis distribution ($P=0.002$) and area of irradiated mucosa showing mucositis ($P=0.028$) were also significant. This indicates that the active pastilles do have a beneficial effect but the magnitude of this is probably smaller than the trial was designed to detect. There was a non-significant trend for patients taking the active pastilles to have less mucosal erythema ($P=0.061$). The incidence of radiation-induced oedema was not significantly different in either arm ($P=0.166$).

The functional consequences of mucositis were significantly reduced in the active arm, severity of dysphagia ($P=0.006$), and especially the highly objective end point, percentage weight loss ($P=0.009$) (Table V). The reduction in pain was just short of statistical significance ($P=0.056$).

Figure 1 shows the number of patients whose mouth washings yielded yeast isolates in each of the weeks after

**Table III** Patient compliance

| *Assessed weeks where patient says that they are still taking pastilles (%)* | *Active* % | *Active* No. | *Placebo* % | *Placebo* No. |
|---|---|---|---|---|
| 100 | 85 | 95 | 76 | 83 |
| 75–99 | 5 | 6 | 14 | 15 |
| <75 | 10 | 11 | 10 | 11 |

**Table IV** Mucositis signs by type of pastille (worst record over assessment period)

| | *Active* % | *Active* No. | *Placebo* % | *Placebo* No. | P-*value* |
|---|---|---|---|---|---|
| Type of membrane | | | | | |
| None/thin | 64 | 72 | 52 | 57 | 0.12 |
| Intermediate/thick | 36 | 40 | 48 | 52 | |
| Type of membrane | | | | | |
| None | 24 | 27 | 11 | 12 | 0.009 |
| Thin | 40 | 45 | 41 | 45 | |
| Intermediate | 33 | 37 | 39 | 42 | |
| Thick | 3 | 3 | 9 | 10 | |
| Erythema of mucosa | | | | | |
| Nil | 0 | 0 | 1 | 1 | 0.060 |
| Slight | 33 | 37 | 24 | 26 | |
| Moderate | 61 | 68 | 62 | 68 | |
| Severe | 5 | 6 | 13 | 14 | |
| Mucositis distribution | | | | | |
| None | 24 | 27 | 11 | 12 | 0.002 |
| Patchy | 62 | 69 | 61 | 67 | |
| Confluent | 14 | 16 | 27 | 30 | |
| Degree of dehydration | | | | | |
| Nil | 95 | 105 | 96 | 102 | 0.681 |
| Slight | 5 | 5 | 3 | 3 | |
| Moderate | 0 | 0 | 1 | 1 | |
| Oedema | | | | | |
| Nil | 56 | 62 | 43 | 46 | 0.174 |
| Slight | 32 | 35 | 44 | 47 | |
| Moderate | 12 | 13 | 12 | 13 | |
| Mucositis area | | | | | |
| Median | 30 | | 40 | | 0.028 |
| IQ range | 5–60 | | 15–70 | | |
| Range | 0–100 | | 0–100 | | |

**Table V** Mucositis symptoms by type of pastille (worst recorded over assessment period)

| | *Active* % | *Active* No. | *Placebo* % | *Placebo* No. | P-*value* |
|---|---|---|---|---|---|
| Pain on swallowing | | | | | |
| None | 13 | 14 | 10 | 11 | 0.056 |
| Slight | 55 | 62 | 40 | 44 | |
| Moderate | 23 | 26 | 38 | 41 | |
| Severe | 9 | 10 | 12 | 13 | |
| Severity of dysphagia | | | | | |
| None | 8 | 9 | 4 | 4 | 0.006 |
| Some discomfort | 30 | 34 | 17 | 19 | |
| Difficulty swallowing | 44 | 50 | 55 | 60 | |
| Considerable difficulty | 17 | 19 | 18 | 20 | |
| Severe difficulty | 0 | 0 | 2 | 2 | |
| Nasogastric tube | 0 | 0 | 4 | 4 | |
| Percentage weight loss (kg) | | | | | |
| Median | 3.3 | | 5.1 | | 0.009 |
| IQ range | 1.4–5.5 | | 2.0–8.5 | | |
| Range | 0.0–14.9 | | 0.0–17.06 | | |

**Figure 1** Yeast isolates during and after radiotherapy.

starting pastille treatment. The treatment group showed a striking and statistically significant reduction in the number of yeast-positive patients for all of the weeks studied. A separate analysis (Table VI) confirmed that the pastilles significantly ($P = 0.003$) reduced the percentage of patients with one or more yeast isolates during treatment and that this result was unaffected by the presence or absence of yeasts in pretreatment cultures.

In contrast, the active pastilles failed to reduce the percentage of patients with AGNB cultured during treatment ($P = 0.256$), irrespective of whether or not these bacteria were cultured before treatment (Table VII). There was a consistent reduction in the proportion of treated patients whose mouth washings yielded AGNB in any one week (Figure 2), but this effect was only seen from week 3 of treatment and reached statistical significance in only 3 of the 7 weeks affected.

The mouth washings yielded a wide range of AGNB species, with a preponderance of coliform strains (Table VIII). Isolates from the pastille and placebo groups showed no significant difference in the distribution of AGNB species.

The relationship of the microbiological results to pseudomembrane formation is of particular interest because pseudomembranes are generally accepted as an index of severe mucositis. Table IX shows that patients who had thick or intermediate pseudomembrane formation during treatment were significantly more likely to have shown cultures positive for AGNB ($P = 0.003$). A similar, but less striking relationship (Table X) was seen with yeasts ($P = 0.026$).

Given the very marked effect of active pastilles on yeast isolates, it might be expected that the relationship of severe mucositis with yeast presence would be weaker in patients receiving active pastilles. (Table XI) confirms that this was indeed the case: for active pastilles there was very little difference in the percentage of patients with intermediate/thick pseudomembranes according to the presence or absence of yeasts (32% *vs* 36%) whereas for the placebo group this difference was large (38% *vs* 52%).

There was no significant association between treatment group and the incidence of resistance among AGNB to the pastille drugs, and this finding was not affected by the detection of resistant AGNB strains in pretreatment samples. However, following the onset of treatment, a significant minority (13%) of all pastille patients showed at least one isolate of a resistant strain (mainly *Proteus* or *Serratia* species resistant to polymyxin). Outgrowth of resistant Gram-positive organisms (including enterococci) was not seen and there were no clinical problems associated with antibiotic resistance.

For both active and placebo pastilles, patient groups with higher treatment compliance had about a 25% smaller incidence of patients with positive yeast cultures. In contrast, compliance had no significant effect on the isolation of AGNB (Table XII). The reduced isolation of yeasts in the placebo group is at first sight surprising. However, it is likely that patients who used the pastilles as prescribed were also conscientious in following advice about oral hygiene and these procedures may have affected the oral flora as revealed in the mouthwash samples. The absence of a compliance effect on AGNB, together with the disappointing effect of active-pastilles against these organisms, suggests that reduction of oral microbial populations by whatever means may be more difficult with AGNB than with yeasts.

**Table VI** Percentage of patients with yeast present during radiotherapy

| | Patients with yeast present during XRT (%) Active | Placebo |
|---|---|---|
| Yeast present before XRT? | | |
| No ($n = 159$) | 30 | 51 |
| Yes ($n = 30$) | 70 | 90 |

($P = 0.003$).

**Table VII** Percentage of patients with AGNB present during radiotherapy

| | *Active* | *Placebo* |
|---|---|---|
| AGNB present before XRT? | | |
| No ($n = 131$) | 46 | 58 |
| Yes ($n = 58$) | 85 | 84 |

($P = 0.256$).

**Figure 2** Aerobic Gram-negative bacilli isolates during and after radiotherapy.

**Table VIII** Oral carriage of AGNB

| | *Number of patients*[a] *Active* | *Placebo* |
|---|---|---|
| *Escherichia coli* | 38 | 28 |
| *Enterobacter* species | 38 | 20 |
| *Klebsiella* species | 29 | 26 |
| *Proteus* species | 7 | 12 |
| *Pseudomonas* species | 17 | 17 |
| Other AGNB[b] | 27 | 35 |

[a]Number of patients from whom an organism was isolated in any of the samples taken. [b]*Acinetobacter, Citrobacter, Hafnia, Serratia* and unidentified species of aerobic Gram-negative bacilli.

**Table IX** Association between AGNB cultures and degree of mucositis

| | *AGNB present during treatment?* *No* (n = *72*) | *Yes* (n = *117*) |
|---|---|---|
| Type of membrane | | |
| None | 26 | 15 |
| Thin | 46 | 39 |
| Intermediate | 26 | 40 |
| Thick | 1 | 7 |

($P = 0.003$).

Table X Association between yeast cultures during treatment and degree of mucositis

| | Yeasts present during treatment? No (n=102) | Yes (n=87) |
|---|---|---|
| Type of membrane | 25% | 13% |
| None | 41% | 41% |
| Thin | 31% | 39% |
| Intermediate | 3% | 7% |
| Thick | | |

($P=0.026$).

Table XI Association between mucositis and yeasts by type of pastille (active or placebo)

| | Active pastilles Yeast present during treatment? | | Placebo pastilles Yeast present during treatment? | |
|---|---|---|---|---|
| | No (n=65) | Yes (n=33) | No (n=37) | Yes (n=54) |
| Type of membrane | | | | |
| None | 28% | 18% | 19% | 9% |
| Thin | 40% | 45% | 45% | 39% |
| Intermediate | 29% | 36% | 35% | 41% |
| Thick | 3% | 0% | 3% | 11% |

Table XII Effect of compliance upon yeast and AGNB cultures during radiotherapy

| | Active pastilles Compliance | | Placebo pastilles Compliance | |
|---|---|---|---|---|
| | <80% (n=8) | ⩾80% (n=90) | <80% (n=10) | >80% (n=81) |
| Yeast present during treatment | 75% | 30% | 70% | 59% |
| AGNB present during treatment | 50% | 60% | 60% | 65% |

### Discussion

Therapeutic radiation dosage is usually limited to levels where the risks of late damage are small. The severity of symptoms caused by acute mucositis may limit the radiation dose given and compromise the chance of cure. There is a steep dose–response curve for most head and neck cancers (Shukowsky and Fletcher, 1973). To obtain optimum results treatment must be given close to normal tissue tolerance. A rest during treatment of 2–3 weeks certainly reduces acute mucosal side-effects, but cure rates are also substantially reduced (Amdur *et al.*, 1989). More severe forms of mucositis seem to develop in certain groups of patients. These include those treated after surgery or receiving accelerated forms of radiotherapy. After surgery, there may be impaired motility of normal structures and grafts may still be healing with areas of necrotic tissue which may be colonised by abnormal microbial flora. Radiotherapy fields tend to be large and include one or more parotid glands leading to reduction in saliva production. Saliva washes away intraoral debris, food particles and bacteria and contains immunoglobin A, all factors important in the maintenance of normal mucosal flora.

There is no standard therapy to prevent or treat radiation mucositis. The use of the antiseptic mouthwash chlorhexidene has been shown to be ineffective (Skijhervet *et al.*, 1989*b*) or even harmful (Foote *et al.*, 1994). Soluble aspirin has been popular for many years as a treatment for this condition but it has never been tested in a randomised controlled trial. It is possible that the anti-inflammatory properties of aspirin are important along with its analgesic effects. Benzydamine, a non-steroid anti-inflammatory agent with anaesthetic and antimicrobial properties was tested in a double-blind placebo-controlled trial containing 43 patients. The degree of mucositis was statistically significantly reduced and there was a non-significant trend in the reduction of radiation-induced symptoms in favour of the active arm (Epstein *et al.*, 1989). Other agents, in particular sucralfate, a coating agent which binds to ulcerated areas, have shown promise in small uncontrolled studies but have not been found to be effective when assessed more rigorously (Epstein and Wong, 1994).

This large, randomised, placebo-controlled, double-blind trial shows significant, beneficial effects of a selective decontamination regimen applied to the buccal mucosa and oropharynx. There was a reduction in mucositis distribution ($P=0.002$), mucositis area ($P=0.028$), dysphagia ($P=0.006$) and especially weight loss ($P=0.009$) which is a highly objective end point. These results support the findings of a small, uncontrolled study when 15 patients were given lozenges containing the same antibiotics (Spijkervet *et al.*, 1991). However, our findings are less striking. In particular, the formation of pseudomembranes was inhibited rather than completely prevented as described by Spijkervet and colleagues.

Pseudomembrane formation is important, both because it is an index of severe mucositis and because it is the principal feature that distinguishes irradiation mucositis from yeast stomatitis. The latter is an acute infection of the buccal mucosa characterised by burning, tenderness or dryness and the presence of white patches or deep red erosions. Pseudomembranes consist of extruded plasma and dead cells and their formation probably results from the combined effects of irradiation damage and abnormal microbial colonisation of the damaged mucosa. Both yeasts and AGNB have been implicated in the microbial component, and it has been suggested that AGNB are the principal factor (Spijkervet *et al.*, 1991; Martin, 1993). The results of this study strongly suggest that yeast colonisation is a major factor in the pathogenesis of the condition. However, they are also compatible with an important role for AGNB, particularly in the formation of the more advanced grades of pseudomembranes. More investigation is required to elucidate the complex microbiological features of this condition.

The significant clinical benefit demonstrated with pastille use probably relates to effects on both yeast stomatitis and irradiation mucositis. Any effects of the pastilles in preventing yeast stomatitis can reasonably be ascribed to the amphotericin B component of the formulation. However, the reduction in pseudomembrane formation may reflect a complex interaction between the marked antifungal effect of the amphotericin B combined with the less striking effect shown by the two antibacterial agents against AGNB.

The high proportion (76%) of patients in the active group who suffered some form of pseudomembrane formation may have resulted from the limited ability of the pastille regimen to reduce oral mucosal populations of AGNB. This disappointing microbiological result contrasts strongly with the use of the same antibacterials applied as a gel or paste to the buccal mucosa in intensive care patients, when AGNB populations are drastically reduced within 3–4 days (Stoutenbeek *et al.*, 1984; Ledingham *et al.*, 1988). The striking success of the latter regimens may well reflect prolonged drug exposure of mucosal organisms resulting from the adherent gel or paste preparations used. This feature, combined with the partial success of pastilles in the present study, suggests a need for new formulations to allow protracted delivery of antimicrobials to the buccal mucosa of ambulant patients.

In spite of these reservations, the present study has demonstrated that the use of pastilles containing tobramycin, polymyxin and amphotericin produced a reduction in mucositis problems that should be of sufficient magnitude to increase materially patient tolerance to radical radiotherapy for head and neck cancer.

**Acknowledgements**
We thank Drs P Canney, M Russell and AG Robertson for entering patients into the study.

### References

AMDUR RJ, PARSONS JT, MENDENHALL WM, MILLION RR AND CASSISI NJ. (1989). Split-course versus continuous irradiation in the postoperative setting for squamous carcinoma of the head and neck. *Int. J. Radiat. Oncol. Biol. Phys.*, **17**, 279–285.

COLLEE JG, DUGUID JP, FRASER AG AND MARMION BP. (1989). *Mackie and McCartney Practical Medical Microbiology*. Churchill Livingstone: Edinburgh.

EPSTEIN JB, STEVENSON-MOORE P, JACKSON S, MOHAMMED JH AND SPINELLI JJ. (1989). Prevention of oral mucositis in radiation therapy: a controlled study with benzydamine hydrochloride rinse. *Int. J. Radiat. Oncol. Biol. Phys.*, **16**, 1571–1575.

EPSTEIN JB AND WONG LW. (1994). The efficacy of sucralfate suspension in the prevention of oral mucositis due to radiation therapy. *Int. J. Radiat. Oncol. Biol. Phys.*, **28**, 693–698.

FOOTE RL, LOPRINZI CL, FRANK AR, O'FALLON JR, GULAVITA S, TEWFIK HH, RAYN MA, EARLE JM AND NOVOTNY P. (1994). Randomized trial of a Chlorhexidene mouthwash for alleviation of radiation-induced mucositis. *J. Clin. Oncol.*, **12**, 2630–2633.

LEDINGHAM IMcA, ALCOCK SR, EASTAWAY AT, McDONALD JC, McKAY IC AND RAMSAY G. (1988). Triple regimen of decontamination of the digestive tract, systemic cefotaxime, and microbiological surveillance for prevention of acquired infection in intensive care. *Lancet*, **1** 785–790.

LEHMAN EL. (1975). *Nonparametrics*. pp. 145. Holden-Day: San Francisco.

MARTIN MV. (1993). Irradiation mucositis: a reappraisal. *Eur. J. Cancer*, **29B**, 1–2.

POCOCK SJ. (1977). Group sequential methods in the design and analysis of clinical trials. *Biometrika*, **64**, 191–199.

SHUKOVSKY SE AND FLETCHER GH. (1973). Time–dose and tumour volume relationships in the irradiation of squamous carcinoma of the tonsillar fossa. *Radiology*, **107**, 621–626.

SPIJKERVET FKL, VAN SAENE HKF, PANDERS AK, VERMEY A AND MEHTA DM. (1989*a*). Colonisation index of the oral cavity: a novel technique for monitoring a colonization defence. *Microbiol. Ecol. Health Dis.*, **2**, 145–151.

SPIJKERVET FKL, VAN SAENE HKF, PANDERS AK, VERMEY A, MEHTA DM AND FIDLER V. (1989*b*). Effect of chlorhexidine rinsing on the oropharygeal ecology in patients with head and neck cancer who have irradiation mucositis. *Oral. Surg. Oral. Med. Oral. Pathol.*, **67**, 154–161.

SPIJKERVET FKL, VAN SAENE HKF, VAN SAENE JJM, PANDERS AK, VERMEY A AND MEHTA DM. (1991). Mucositis prevented by selective elimination of oral flora in irradiated head and neck cancer patients. *J. Oral. Pathol. Med.*, **19**, 486–489.

STOUTENBEEK CHP, VAN SAENE HKF, MISANDA DR, VAN DER WAAIY DF AND ZANDSTRA DF. (1984). The effect of selective decontamination of the digestive tract on colonisation and infection rate in multiple trauma patients. *Intensive Care Med.*, **10**, 185–192.

VAN SAENE HK AND MARTIN MV. (1990). Do microorganisms play a role in irradiation mucositis? *Eur. J. Clin. Microbiol. Infect. Dis.*, **9**, 861–863.

WHITEHEAD J AND BRUNIER H. (1989). *Planning and Evaluation of Sequential Trials*. Department of Applied Statistics, University of Reading.